# Process evaluation of the Teamplay parenting intervention pilot: implications for recruitment, retention and course refinement

**DOI:** 10.1186/1471-2458-13-1102

**Published:** 2013-12-01

**Authors:** Russell Jago, Simon J Sebire, Georgina F Bentley, Katrina M Turner, Joanna K Goodred, Kenneth R Fox, Sarah Stewart-Brown, Patricia J Lucas

**Affiliations:** 1 Centre for Exercise, Nutrition & Health Sciences, School for Policy Studies, University of Bristol, Bristol, UK; 2 School of Social and Community Medicine, University of Bristol, Bristol, UK; 3 Warwick Medical School, University of Warwick, Coventry, UK; 4 Centre for Research in Health and Social Care, School for Policy Studies, University of Bristol, Bristol, UK

**Keywords:** Parenting, Recruitment, Retention, Intervention, Process evaluation

## Abstract

**Background:**

Parenting programs could provide effective routes to increasing children’s physical activity and reducing screen-viewing. Many studies have reported difficulties in recruiting and retaining families in group parenting interventions. This paper uses qualitative data from the Teamplay feasibility trial to examine parents’ views on recruitment, attendance and course refinement.

**Methods:**

Semi-structured interviews were conducted with 16 intervention and 10 control group parents of 6–8 year old children. Topics discussed with the intervention group included parents’ views on the recruitment, structure, content and delivery of the course. Topics discussed with the control group included recruitment and randomization. Interviews were digitally recorded, transcribed and thematically analyzed.

**Results:**

Many parents in both the intervention and control group reported that they joined the study because they had been thinking about ways to improve their parenting skills, getting ideas on how to change behavior, or had been actively looking for a parenting course but with little success in enrolling on one. Both intervention and control group parents reported that the initial promotional materials and indicative course topics resonated with their experiences and represented a possible solution to parenting challenges. Participants reported that the course leaders played an important role in helping them to feel comfortable during the first session, engaging anxious parents and putting parents at ease. The most commonly reported reason for parents returning to the course after an absence was because they wanted to learn new information. The majority of parents reported that they formed good relationships with the other parents in the group. An empathetic interaction style in which leaders accommodated parent’s busy lives appeared to impact positively on course attendance.

**Conclusions:**

The data presented indicate that a face-to-face recruitment campaign which built trust and emphasized how the program was relevant to families positively affected recruitment in Teamplay. Parents found the parenting component of the intervention attractive and, once recruited, attendance was facilitated by enjoyable sessions, empathetic leaders and support from fellow participants. Overall, data suggest that the Teamplay recruitment and retention approaches were successful and with small refinements could be effectively used in a larger trial.

## Background

Physical activity (PA) is associated with lower levels of several cardio-metabolic risk factors and improved mental well-being among young people [1-4]. Screen-viewing (SV) is an independent behavior which has also been associated with increased risk of obesity and poorer mental well-being [5,6]. Many children exceed current SV recommendations [7-14] and do not engage in sufficient amounts of PA [15-17]. Thus, interventions that focus on increasing PA and reducing SV among children and adolescents are needed. The early school years (6–8 years of age) are a key period when children’s PA and SV behaviors are established [5]. This therefore, is a key age to target PA and SV interventions. Group parenting courses have been widely used to change a number of behaviors including drug use, anti-social behavior and obesity treatment [18-23]. There is however, a shortage of research that examines the utility of parenting courses for changing children’s PA and SV.

We have recently reported on the results of a feasibility trial evaluation of a new PA and SV parenting course called “Teamplay” [24]. The evaluation of Teamplay showed that the intervention had the potential to yield a 10-minute per weekend day increase in the minutes of moderate-to-vigorous PA of both 6–8 year old children and their parents. The study also showed the potential to positively affect the SV behaviors of both children and their parents.

In this paper, we report results of the process evaluation of the Teamplay feasibility trial. Of particular interest in the process evaluation were participants’ views on recruitment to the intervention, reasons for joining and factors which facilitated and hindered attendance. A number of parenting courses have reported difficulties in recruiting and retaining participants [25] yet these factors are critical to the success of any behavioral intervention. A recent review [26] of factors associated with poor attendance and engagement with parenting support courses suggests that a number of separable but inter-related factors are associated with attendance including: personal life, structural, perceptual and course factors [26]. It is not clear, however, how these categories may apply to parenting courses that focus on children’s PA and SV or what the effective strategies to overcome these issues might be. Further, from a research perspective, it is essential to understand participants’ views about participation in a research trial with the knowledge that they will be randomly allocated to the intervention or control arm (both to increase likely participation and reduce potential bias in participation). Accordingly, we explored the views of parents in both intervention and control arms regarding their experience of the randomization process. We also sought to evaluate parents’ views in the intervention group about the course structure, the content covered, the perceived usefulness of each session, and the leader delivery style.

The specific research questions that this paper was designed to address were: 1) What factors affected participants’ decisions to join the study and how could recruitment be improved?; and 2) How could the parenting course be improved? The implications of the findings are then presented in relation to potential strategies that could be implemented to increase recruitment and retention in PA and SV parenting courses and to further develop interventions similar to Teamplay.

## Methods

### Study design

The design and content of the Teamplay intervention have been reported in detail elsewhere [24]. Briefly, Teamplay was a two-arm randomized controlled feasibility trial. Participants were parents of 6–8 year old children who were recruited via leaflets and advertisements that were distributed across the community in coffee shops, children’s centers, play groups, school playgrounds and community centers and face-to-face in schools and the community [24]. Participants were invited to attend an eight week parenting course consisting of a weekly two hour session. Participants were recruited from two neighborhood in the City of Bristol (UK). One neighborhood was selected from the lowest (low Socio Economic Status - SES) and one from the middle (mid SES) tertile of deprivation according to the index of multiple deprivation (IMD) [27] for the city of Bristol. A total of three courses were run: 1) daytime in the low SES neighborhood; 2) daytime in the mid SES neighborhood; and 3) a mixed SES evening course drawing from both neighborhoods. A free crèche was provided for parents who attended the daytime courses.

Intervention sessions focused on increasing child PA and reducing SV by promoting active play, supporting parents to set boundaries and goals, and by demonstrating games which built physical skills in parents and children. The intervention was based on self-determination theory (SDT) [28] and included a detailed leader manual. The course was delivered by two members of the research team (GFB and JKG) who had received Parent Group Leader training from Family Links. The two team members had postgraduate training in PA, had extensive experience of delivering PA courses but were not parents. At the end of each session, parents were provided with “Put into Practice” materials which aimed to encourage parents to practice the skills learnt in the session at home with their children. The intervention yielded an increase of around 10 minutes in the children’s minutes of moderate-to-vigorous PA per day at the weekend with comparable increases among the parents [24]. Weekly attendance ranged from 52% to 84% [24].

The data reported in this study include process evaluation data collected from interviews with participants from the intervention and control arms, and questionnaire data that were completed at the end of each Teamplay session.

### Interviews

#### Sampling

Interviews were conducted with 16 parents who took part in the intervention. The sample included 8 who had attended the daytime course (4 mid SES, 4 low SES neighborhoods), and 4 parents from the evening course. Participants were purposefully selected based on attendance level so that interviews would represent parents with varying levels of adherence. Ten parents from the control group were contacted at the same time as the intervention parents (2 parents recruited to the daytime mid SES neighborhood control group, 2 from the daytime low SES control group, and 6 parents from the evening control group).

#### Interview methods

Interviews were chosen as the primary means of data collection as we wanted to provide participants an opportunity to confidentially share their views on the key research questions without being influenced by the views of other participants. To encourage the participants to freely share their views on the program, the interviews with the intervention parents were carried out by a researcher who was independent of the project. Due to funding limitations, and the minimal contact between the research staff and the control group the control group interviews were carried out by the two researchers who had delivered the intervention. Separate semi-structured interview guides were used for the intervention and the control group interviews. Topics discussed with the intervention group included parents’ views on the recruitment, structure, content and delivery of the course, and challenges to attendance. Topics discussed with the control group included parents’ feelings about the recruitment and randomization. All interviews were administered within a month of the end of the intervention period between March and June 2012.

Data collection continued until data saturation was deemed to have been reached. To aid communication, six parents from the intervention group, for whom English was not their first language, were interviewed face-to-face with all other interviews conducted by phone. All interviews were digitally recorded, transcribed verbatim and anonymised. Intervention interview duration ranged from 15 to 58 minutes (mean 29.2 minutes). Control group interview duration ranged from 8 to 13 minutes (mean 11.1 minutes). The study was approved by the School for Policy Studies ethics committee at the University of Bristol and written informed consent was obtained for all participants.

#### Session evaluations

At the end of sessions 1–7, all attending intervention participants were asked to rate the usefulness of each of the five elements which that session was designed to include (e.g., Session 1: How useful were the following in today’s session: the welcome; group activity; introduction to PA; building blocks of PA; and the overall view) using a scale ranging from 1 (*terrible)* to 5 (*excellent).* Due to technical issues no data were collected for session 4 at the mid SES location.

#### Analysis

The interview data were analyzed thematically [29]. The approach involved the team reading and re-reading the interview transcripts in order to develop a coding frame and identify emerging themes. To assess the appropriateness of the coding frame, GFB, JG and KMT independently coded three interview transcripts. They then met to discuss their coding. Discrepancies in coding led to the coding frame being revised with new codes being developed and existing codes either being deleted or defined more clearly. GFB and JG then coded some further transcripts. Confident that the coding frame was now appropriate, transcripts were then imported into NVivo etc. (Version 9.0, QSR, Southport, UK) to allow for electronic coding and retrieval of data. To assist with the systematic interpretation of the data, a framework approach was used [30]. A matrix was created using headings from the thematic coding frame, with each participant’s views and experiences summarized within this matrix, across all themes. Quotes reproduced in this paper have been labeled to indicate interviewee gender, trial arm allocation (intervention or control), and intervention group (mid SES, low SES or evening). For the mixed SES evening group we have also identified whether they were recruited from the low or mid SES area.

Quantitative session evaluation responses were summed, aggregated and graphically presented to provide the mean perceived usefulness of each of the seven sessions for the three different intervention delivery locations.

## Results

Nine broad themes emerged during the interviews: A) Reasons for joining the study; B) Understanding of the randomization process; C) Feelings about the first session; D) Reasons why participants kept attending; E) Factors that made it difficult to attend the course; F) Parent’s views of the Teamplay course and how it could be improved; G) Perceptions of the course leaders; H) Course content and delivery and I) Course structure. A summary of the key themes and sub-themes that emerged from the interviews are presented below. Themes related to recruitment are presented for the intervention and control group participants combined because they were asked they same questions and there was little variation in responses. Moreover, participants were recruited before they knew their arm allocation and as such their experiences if recruitment would not be influenced group arm allocation. All other themes are presented separately.

### A). Reasons for joining the project (intervention and control group interviews)

Parents gave a variety of reasons for joining the Teamplay project. Some parents had been thinking about ways to improve their parenting skills, getting ideas about how to change PA and SV behavior, or had been actively looking for a parenting course but with little success in enrolling on one. For these parents, the course came at the right time:

*“It’s good because, you know, we did Peep Squeak [*course for younger children*] when they were little but I think things change as they get a little bit older and I was kind of like thinking you know, I was thinking what can we you know do to improve? It came at the right time really, all the little ideas have really helped.” (Mother, Intervention, mid SES)*

“I mean because prior to doing that I spoke to the health visitor and tried to get on a few parenting courses, one wasn’t running any more, the other one wasn’t in my area and it was difficult you know. I actively went looking for it and I couldn’t get it.” (Mother, Intervention, Evening - mid SES)

#### Project communication

Many of the reasons parents gave for signing up to the Teamplay project related to how effectively the project was communicated to parents initially. The initial promotional materials were distributed via community venues and schools, including in person distribution at school gates. The indicative course topics resonated with some parent’s experiences and represented a possible solution to the parenting challenges they faced:

“We’ve actually signed up for it because it said, ‘does your child have too much energy?” (Mother, Intervention, Evening – mid SES)

“… there are several courses that we get advertised in the newsletters and stuff like that at school, this is the first one that I’ve applied to because it did sound like it might be really suitable for the child that I was doing it with.” (Mother, Control, low SES)

#### Childcare provision

The free daytime creche was viewed positively and the parents believed that this service would encourage parents to attend the course.

“It took her a week or two you know to get settled but the crèche facilities were brilliant.” (Mother, Intervention, Mid SES)

“The daytime ones could have a crèche and that might take the kids away and so the mums are more likely to turn up if they’d got childcare.” (Mother, Intervention, Evening - mid SES)

#### Friend encouragement

Other reasons for joining included being encouraged by a friend who had already signed up to the project, being interested in helping with a research project, and being motivated by the opportunity for peer learning from other parents.

“My friends, she told me… Yeah she told me you enjoy it, starting next week called Teamplay and I say yes I start.” (Mother, Intervention, mid SES)

“I thought actually this is all part of this bigger project that we can contribute so let’s go for it.” (Mother, Control, Evening - mid SES)

“I know when I first talked to you about it I was quite excited about going to a workshop, I think that adds more to it especially as you get to meet other parents and talk to them as well.” (Mother, Control, Evening - low SES)

#### Initial reticence

Teamplay did not appeal initially to all parents who eventually enrolled. The example below shows how one parent’s first impression changed after an informal face-to-face meeting with one of the research team during the recruitment phase:

“Well my initial thing was something came through and I thought oh no I can’t be bothered with that, that’s not me and then a very nice person spoke to me in the playground and gave me a leaflet and I thought oh actually you know this would be quite interesting.” (Mother, Control, Evening - mid SES)

Despite efforts to make descriptions of the course inclusive, this was not always successful. One parent had discussed the course with other parents, and found they had assumed that course would be for parents with problems.

“Some of them seemed to think it maybe was for people with problem children… or, or obese children.” (Mother, Intervention, Evening - mid SES)

### B). Parents’ understanding of the randomization process

The majority of parents understood the randomization process and felt that it was explained clearly.

“Yeah, I mean it (leaflet) explained everything, I didn’t need to ask any questions.” (Mother, Control, Evening - low SES)

No, it was all perfect, perfectly easy to understand and enough information.” (Mother, Control, low SES)

However, some parents felt it wasn’t clear from the first contact that the course was part of a research project and that they would be randomly assigned to one of two groups.

“Not necessarily no, no that wasn’t that clear.” (Mother, Control, Evening).

“Yeah when I first signed up I knew but when I first went along to the … maybe it was the very first flyer I picked up where I thought that would be good I’d like to go to that course, it wasn’t until I found out, whether there was another leaflet, I’m not sure, or when it was signing up, that it was actually a study and you might get on it, or you might not if that makes sense, it wasn’t till a bit further down the line that there were two groups.” (Mother, Control, Evening - mid SES)

### Intervention group

#### C). Feelings about the first session

Although some parents reported having no concerns about attending the first sessions, the majority of parents recognized that factors related to attending a course for the first time, such as meeting new people, and knowing the type of people who will constitute the group could be daunting*.*

“You just want to get that first half hour over with so you know who else is joining” (Mother, Intervention, low SES)

“I think maybe like the first couple of sessions it was like all a bit daunting, you feel a bit sort of conscious but I think once you got to know everybody and got into it, it was quite fun really.” (Mother, Intervention, mid SES)

#### The role of course leader in making participants feel comfortable

The course leaders played an important role in helping parents to feel comfortable during the first session, engaging anxious parents and their friendly approach put parents at ease.

“I thought it was a lot of effort into putting everyone at ease” (Mother, Intervention, Evening - mid SES)

“There was one lady in the course that once you know suffered from a bit of anxiety and one of the girls did get her to go in… Yeah, [leader’s name] she went to the crèche and just sort of said come along and I think just seeing her familiar face and that … if she hadn’t have done she probably wouldn’t have come back”. (Mother, Intervention, low SES)

Meeting the course leaders before the first session was an important factor in helping parents feel more comfortable about joining a new group.

“We also knew the faces of the girls that were running the course because they were the ones that went and talk about the course and used to doing it. So it was good to see a familiar face.” (Mother, Intervention, low SES)

Parents also valued a group activity around setting group rules which helped them to feel comfortable in the group.

“The fact that we were at the beginning discussing all the things helped to make people think about oh this is a nice group and I am going to get along with these people” (Mother, Intervention, low SES)

#### D). Why did parents keep attending?

The most commonly reported reason for parents returning to the course was because they wanted to learn new information. There was a sense that parents did not want to miss out on the upcoming topics which were advertised by the course leaders.

“I had the plan of what was to come in the future and keen not to miss out on certain parts of it.” (Mother, Intervention, Evening - mid SES)

“…they gave us a rundown of what the course was and some of the things sort of later on in the course I thought ‘well that will come in handy” (Mother, Intervention, mid SES)

#### Enjoyment

Some parents reported looking forward to attending because they were enjoying the course with other parents:

“Yes I enjoyed it. If I’m not enjoying it I’m not coming! … Yeah, If I enjoy I give my time yeah.” (Mother, Intervention, mid SES)

*“…it was a nice time, you know we all looked forward to going actually so it was quite good” (Mother, Intervention, Evening - mid SES*)

Others parents referred to their commitment to attending a course and because they felt a responsibility to attend once they had committed:

“If I miss I feel so bad.” (Mother, Intervention, mid SES)

“I think people went to a lot of effort to put that on… I’m like that, I’m quite a reliable person … it’s just my natural personality that just I wouldn’t like to let people down like that.” (Mother, Intervention, Evening - mid SES)

#### Project support

Several parents talked about additional factors which supported their attendance, such as support provided to parents who missed a session, reminder text messages, the convenience of the location or their child’s enjoyment of the crèche.

“Well I spoke to them before and I explained that I couldn’t attend the sessions, and they were very useful because they gave me the material… they sent the material to me so when I went to the next session they discussed with me the session… I feel sorry I didn’t, but at the same time I didn’t need anything because they were very good to feed me back with whatever they did on the sessions” (Mother, Intervention, low SES)

*“If you’re trying to do so much organization for the kids and what you have to do that little reminders* [are] *quite good actually”(Mother, Intervention, low SES)*

#### Refreshments

Parents also appreciated the provision of snacks and facilities to make a drink.

“It was nice having that little kitchen area where you could make a cup of tea.” (Mother, Intervention, Mid SES)

“No I think they made you feel very welcome you know there was like nibbles provided every night because they realized people were coming straight from work” (Mother, Intervention, Evening - mid SES)

#### E). What made it difficult to attend?

Parents talked about factors that hindered their attendance. The main reason for not attending was due to the competing priorities of family life, such as illness or other commitments.

“I don’t think I had any intention of missing any, it was only through a clash with something else that was already arranged so in terms of keep on coming back that was all fine.” (Mother, Intervention, Evening - mid SES)

“the only reason I wouldn’t have gone is if any of us were ill you know or if we had a you know a sort of an appointment already booked.” (Mother, Intervention, mid SES)

Balancing the course with working life was also a factor that affected attendance of some parents:

“It’s a very tricky time of the day trying to get home from work and … get the kids”. (Mother, Intervention, Evening - mid SES)

Parents recognized the impact of the half term school holiday on the following week’s attendance.

“I think that was after the school holiday… We sort of a couple of weeks off, and then I totally forgot about it,.” (Mother, Intervention, low SES)

“I think it was good to have a break in between although I think a few people didn’t turn up after the break, so I don’t know if that, you know if that was a benefit to everybody but it was fine for us” (Mother, Intervention, mid SES)

### F). Parent’s views of the Teamplay course

#### The facilitating group environment - “You weren’t on your own”

The number of parents attending sessions varied between courses [24], but there seemed to be a general sense that between 8 and 10 parents per session was desirable. The Mid SES morning course had the highest and most stable attendance (between 7 and 10 parents per session). All parents interviewed from this course were happy with the group size and thought that it facilitated discussion with other parents. Some parents cautioned against having larger groups as this may discourage individual contributions to discussions. Parents also referred to the relationship between the group size and developing personal bonds with a “core” of regular attendees.

“there were a couple you know that started off and then didn’t complete the course for one reason or another but there was like a core of us that sort of came every, you know, came every week. And yeah it was … you know a small enough group to be sort of quite personal, yeah I think it was just about the right size.” (Mother, Intervention, mid SES)

#### Irregular attendance

The low SES morning course had very inconsistent attendance. In comparison to the idea of the “core” group of frequent attendees, parents identified that irregular attendance was somewhat disruptive to the flow of the sessions. It was suggested that more parents should be recruited initially to allow for attrition and fluctuation in attendance.

“I think maybe 15 [parents on a course], because with every course that starts you’re going to have drop out anyway, so that will allow for a few to drop out and then you’ve still got your 10.” (Mother, Intervention, low SES)

“I thought it was a good number, I think at the beginning, I can’t remember how many there were but maybe ten or twelve. The problem was that sessions were not consistent in the number of people attending.” (Mother, Intervention, low SES)

The evening course had low, but consistent attendance. Only 4 participants were enrolled on this course, plus one participant’s husband who also attended but was not involved in the measurement component of the study. The majority of parents from this course believed that the group was too small and that it would have benefitted from being larger with specific reference to having a greater number of parent’s experiences to share and learn from.

“Yes, I think it would have been better to have more [parents]. I mean we all, a couple were two of the five so it meant there weren’t that many families to talk about different situations and sharing different experiences. So I think more would definitely have been beneficial.” (Mother, Intervention, Evening - mid SES)

#### Relationship with other attendees

The majority of parents reported that they formed good relationships with the other parents in the group. Many reported that sharing experiences with parents of a similar background helped them to bond as a group.

“most of the parents’ (children) went to the same school as my kids so they sort of would, you know, be from a similar area, background so that was … they were very easy to talk to, and the other mothers were very easy to talk to and quite similar to me as well, so probably not what I was expecting seeing as I kind of thought maybe it was for problem families. “ (Mother, Intervention, mid SES)

“And in that course, I enjoyed it because a lot of the mums were the same, you know… So you weren’t on your own if you like, an example used, a lot of the mums say oh yes that would happen with us so you didn’t feel oh god you are just a failure on yourself, you know.” (Mother, Intervention, Evening – mid SES)

### G). Perceptions of the course leaders - “They were so full of enthusiasm”

The majority of parent’s responses regarding the leaders were positive and the course leaders appeared to have made an important impact on parents’ attendance and enjoyment of the course.

“The tutors are the ones that make the course interesting… they make fun out of… you don’t even feel like it’s a lesson you’re learning. (Mother, Intervention, mid SES)

Parents felt that the two course leaders worked well together and were organized. Their friendly and relaxed approach helped to put people at ease and feel part of the group. Parents also valued the leaders’ interaction with parents and the fact that they joined in with activities.

“They both would be part of the discussion and part of the games or activities so I thought that was quite good … the way they involved people to be part of the course and feedback their own experiences was very useful.” (Mother, Intervention, low SES)

An empathetic interaction style appeared to impact positively on course attendance as the leaders accommodated parent’s busy lives and competing demands:

“I have the feeling that because they were so nice, even if I am going late, I still go because they are not going to look at me like why are you here, you know I feel they would really respectful and they were considering people who were attending and understood that people get stressed having a family life” (Mother, Intervention, low SES)

Leader enthusiasm and commitment was also important, often it helped parents to engage and made the course interesting.

“…they were so full of enthusiasm it was quite contagious” (Mother, Intervention, Evening - mid SES)

“It was always like you know this is really important, it felt almost as important to them as it did to you, and it's your family life do you know what I mean so .… that was really good.” (Mother, Intervention, Evening - mid SES)

Based on their perception of the leader’s parenting experience, some parents attending the evening course described their initial reservations about the leaders. Two parents said their initial reservation changed once they got to know the leaders. This finding suggests that initially the parents wanted experiential expertise (i.e., from a mother) but were surprised that the professional expertise from non-mothers was still useful, thereby indicting that professional expertise is helpful, provided the session leader has the skills needed to run these groups.

“Oh, I think they were lovely, they did a great job. At the start I was kind of thinking, you know, they don’t look like they have [children]… they, they don’t really look like they know much about… the sharp end of parenting”. (Mother, Intervention, Evening mid SES)

“At first I was a bit dubious because oh god they are so young and haven’t even got children you know but that stopped, you know you stopped feeling that way once you got to know them because obviously they had done all their training and they knew.” (Mother, Intervention, Evening - mid SES)

One parent felt that parental experiences would have been more beneficial.

“It’s a bit tough to say you’ve got to be a parent to run the course but I think there are valuable experiences which are helpful.. I think if you are including parenting techniques in it, yes that would be my only kind of query.” (Mother, Intervention, Evening - mid SES)

### Course content and delivery - “It covered everything you needed to know”

Parents reported that they were happy with the overall concept of Teamplay, they felt that the three components of parenting, PA and SV were clearly connected and that the course progressed logically.

“Yeah I think, I think the course ran really well and it was like a natural flow really.” (Mother, Intervention, mid SES)

“No I think I was happy with it all actually and like I said it all seemed to balance out really well and make the big picture.” (Mother, Intervention, Evening - mid SES)

#### Topic number and coverage

Most parents felt that the course covered all the content that they required.

“It covered everything I think you needed to know… it gave you all the tools to be able to handle most situations that occurred.” (Mother, Intervention, Evening - mid SES)

Some parents felt the number of topics covered was too much and often content was rushed in order to fit everything in. Some parents would have liked to have spent more time on particular topics.

“I understand that because of the amount of things you are covering you try to do the maximum amount in the time but sometimes I thought on some topics deserved a bit more time.”(Mother, Intervention, low SES)

Parents valued the peer interaction and discussions with other parents through pair and group activities. This facilitated peer learning and parents enjoyed the social aspect of talking with parents.

“I enjoyed interaction between the different members of the group and also between the two persons that were facilitating the sessions.” (Mother, Intervention, low SES)

“Everybody talking, ideas, more ideas…” (Mother, Intervention, mid SES)

“when they were chatting and you know sort of taking stuff in you don’t have chance to sort of think ‘oh god this is boring’ you know. It was just the right you know sort of mix to keep your attention span as well. “(Mother, Intervention, mid SES)

#### Content length

Whilst parents valued this style of delivery, some parents attending the low SES course felt that discussions were too lengthy, which disengaged parents or meant that subsequent topics were hurried or neglected.

“Yeah, I think it could have done with a little bit longer, especially in our group because people seemed to like to talk….” (Mother, Intervention, low SES)

“… during many sessions the talk, the discussions were carried on… Because sometimes no, you could feel a bit bored as well, you know, when it goes on and on.” (Mother, Intervention, low SES)

Whilst some parents felt the ‘Put into Practice’ component (trying things out at home) was an important part of the course, others said that being busy at home prevented them from engaging fully with the take home materials.

“even if we haven’t done it yet it’s not like we’ve given up or anything, it’s not a very good excuse is it but busy lives.” (Mother, Intervention, Evening – mid SES)

“Yeah, yeah, I know it is good because otherwise you could be just going there, taking the handouts, coming back not thinking about it at all until next.” (Mother, Intervention, low SES)

#### I). Course structure

Overall, parents in both the morning courses and the evening course felt that the timing of the weekly session and the length of the course was suitable and convenient. However, many parents also reported that they would have liked the session or the course length to have been longer, because they were enjoying the course, or to allow more time for discussion.

“I think it’s okay but for me it was two hours and a half maybe, because when you spend two hours and a half and then you have a good discussion or someone is telling you about her experience or his experience it’s like you need to have more time to listen to what she wanted to say.” (Mother, Intervention, mid SES)

“Yes, yes so I suppose … perhaps an extra week or two might have given a bit of chance to think about all the different techniques a bit more.” (Mother, Intervention, Evening - mid SES)

However, another parent mentioned that having a longer session would be problematic due to competing priorities. A number of parents on the evening course said that initially they felt that 8 weeks was a long time, which suggests that having a longer course may be off-putting to parents at the stage of recruitment.

“No, two hours is good. Yeah no more. I’m mum … I need cooking, cleaning.” (Mother, Intervention, mid SES)

*“Well at the beginning it sounded a lot and there was a two week break for Easter …which again it made it seem, it seemed like it went on for a long time. But because I was quite into it I didn’t mind at all. Had it, had I not been getting anything from it I think it would’ve been quite long*.” *(Mother, Intervention, Evening - mid SES)*

### Perceived usefulness

**Figure 1:**
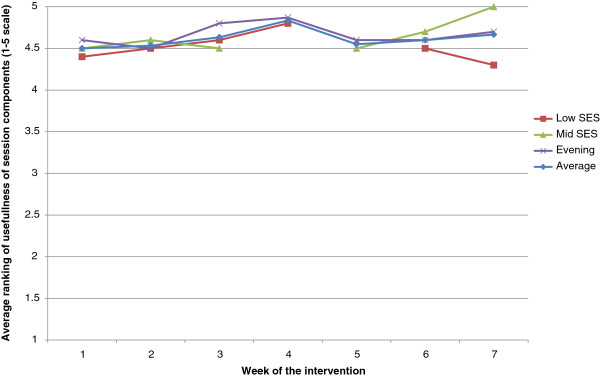
Perceived usefulness of Teamplay content by week of intervention.

The mean perceived usefulness of sessions 1–7 is presented by intervention delivery location in Figure 1. The figure indicates that on a scale that ranged from 1–5 the mean perceived usefulness was on the average above 4.5 for all sessions.

## Discussion

The data reported in this paper indicate that there were three main reasons participants had joined the study: 1) interest in the topic area and relevance to their family; 2) encouragement from friends; and 3) face-to-face recruitment via a member of the project team who would be delivering some of the content. These reasons for joining the study are consistent with results from a previous general parenting course where parents reported seeking out a parenting course, and being ready to make the necessary changes [31]. It is of interest however that the parenting component of this course was specifically mentioned as attractive. Previous research has suggested that peer support is helpful in encouraging adults to join and attend weight loss courses [32,33] and the data presented here reinforce those findings by suggesting that group peer support is also likely to positively affect recruitment into parenting courses. These findings indicate that stressing the potential relevance of the project to families and the main focus of the intervention in recruitment materials is likely to resonate with parents’ situations and encourage them to join the study.

Participants in both the intervention and control groups reported that the face-to-face recruitment method was an important factor in their recruitment to the intervention. Previous research concerning parenting courses has reported that recruitment is difficult [34] with low levels of recruitment adversely affecting statistical power in trials. The face-to-face approach that we adopted might be an effective way of overcoming this issue but it is important to recognize that this approach requires significant investment of time and human resource.

It is important to note that some parents developed an incorrect perception of the intervention being aimed exclusively at obese children or “problem children”. As such, it is imperative that recruitment materials are clearly worded to avoid misperceptions and ensure that potential participants are aware of what they are signing up for at the point of recruitment.

The perceived usefulness of and interest in the Teamplay content were two factors that appeared to positively affect retention. This finding is consistent with a recent review of parenting support interventions [26] which found that course factors including content, delivery and organization are likely to be important factors in retention once participants have been enrolled into the intervention. Thus, it appears that the content, which was informed by extensive formative research, [24,35] was a factor which encouraged participants to attend.

Parents’ reasons for continued attendance centered on their enjoyment of sessions and the social support provided from fellow participants and group leaders including providing refreshments and opportunities for informal conversations. The empathetic leader style which accommodated the busy lives of parents (i.e., allowing late arrival to sessions) appears to have removed a potential barrier to attendance. These findings are consistent with previous parenting research which have stressed the importance of adopting a non-stigmatizing approach [36,37] and good relationships between the parent and trainer [38,39].

Participants in the intervention groups were parents who regularly attended, identified that a strong group ethos or “core group” perception underpinned their enjoyment and facilitated helpful peer-learning within sessions. Both the content of the intervention and the social environment created by the leaders was informed by SDT [28,40]. Specifically we sought to encourage parents to use autonomy-supportive motivational strategies and interactions with their children through the intervention environment which itself embraced the same principles (i.e., delivering fun and enjoyable session, supporting parent-parent relatedness, offering choice /, being empathic, and providing early opportunities for success). The findings related to parent enjoyment and of the facilitating influence of strong social connections within intervention groups is conceptually consistent with SDT, which posits the beneficial behavioral and well-being effects of perceptions of belonging and intrinsic motivation for activities, which require behavioral investment (i.e., attending a parenting course) [28,40]. In addition, the structural support that was afforded via the free crèche was identified as a key variable that positively enhanced the participant’s ability to attend the sessions. Importantly, the participants also indicated that the main reason for non-attendance was competing priorities such as work or family commitments which hindered attendance. Collectively, these findings suggest that focusing on SDT consistent strategies to promote enjoyment and social aspects of the course is likely to be helpful in both recruiting participants to group-based parenting interventions and retaining the participants once the intervention has been established.

### Implications for the course improvement

Although the feedback on the overall content of the intervention was positive, the process evaluation highlighted some content-related issues that might have affected retention which warrant further discussion. Firstly, although accommodating irregular attendance removed a potential barrier to attendance, it was clear that inconsistent attendance in some intervention groups adversely affected the group experience. This suggests that a balanced approach to attendance is needed which emphasizes the importance of regular attendance with the realities of competing parental priorities. One way to address this might be to allow parents to agree on realistic ground rules of attendance in the first session guided by leaders who emphasize the importance of developing a group identity. Parents also reported that attendance was encouraged by knowing the content of forthcoming sessions. This could be emphasized more and flexibility within the session plans could provide opportunities to address parent-requested topics.

Secondly, some of the intervention participants felt that too much content was delivered in the 8-week course, resulting in covering some topics too quickly. This issue could be addressed by: 1) increasing the course duration; 2) removing content; or 3) re-organizing content in response to key issues raised by participants; or 4) managing group discussions so that they use less time. Each solution presents a challenge, but all are relatively minor changes would require only small edits to the intervention manual.

### Strengths and limitations of this study

The current study has provided helpful information on the lessons learnt from the Teamplay feasibility trial, including the factors that affected recruitment and retention. It is important, however, to recognize that the study has a number of limitations. Firstly, the study involved a relatively small number of participants which limits our ability to generalize the findings beyond the current study and setting. However, saturation was reached in both the intervention and control groups and as such we feel that these findings provide a good overview of the views of the intervention and control group participants. It is also important to highlight that the study purposely recruited families from middle and low SES areas of Bristol and as such we have no evidence of how these findings might relate to participants from higher socio-economic groups. The perceived usefulness data were missing from one of the intervention groups and there is a degree of incomplete data. Finally, and perhaps most importantly, the current study provides no information on the participants who did not consent to take part in the study and therefore we cannot be sure about why these potential participants did not take part in the Teamplay project.

## Conclusions

The data presented in this paper indicate that a face-to-face recruitment campaign which built trust and emphasized how the program was relevant to families positively affected recruitment into the Teamplay feasibility trial. Some parents who agreed to take part in the trial were attracted by the parenting component of the intervention. Once recruited, retention was facilitated by enjoyable sessions, empathetic leaders and support from fellow participants. The “no pressure” approach to attendance also facilitated attendance but needs to be managed carefully to prevent adverse effects on group dynamics. Overall, data suggest that the Teamplay recruitment and retention approaches were successful and with small refinements could be effectively used in a larger trial.

## Competing interests

Professor Stewart-Brown is a Trustee of Family Lives a charity which supports parenting interventions. No other authors have any conflicts of interest to declare.

## Authors’ contributions

The study was conceived by RJ, SJS, KMT, SSB, KRF and PJL who secured funding. The qualitative data were analyzed by GFB, JKG and KMT. RJ produced the first draft of the paper with all other authors providing sections and critically reviewing the paper. All authors approved submission.

## Pre-publication history

The pre-publication history for this paper can be accessed here:

http://www.biomedcentral.com/1471-2458/13/1102/prepub
